# Genome-Wide Identification and Characterization of the JMJ Histone Demethylase Gene Family in Maize (*Zea mays* L.) and Its Potential Role Under Drought Stress

**DOI:** 10.3390/biology15070534

**Published:** 2026-03-27

**Authors:** Li Gao, Hui Tian, Xiangli Bai, Aokun Shi, Mian Wang

**Affiliations:** 1 School of Agriculture, Liaodong University, Dandong 118003, China; tianhui@liaodongu.edu.cn (H.T.); 922031@liaodongu.edu.cn (X.B.); 2 Beijing Yunxin Yuetian Technology Co., Ltd., Beijing 100081, China; 13241487789@163.com (A.S.); 13470007290@163.com (M.W.)

**Keywords:** maize, JMJ gene family, histone demethylation, phylogenetic analysis, drought stress

## Abstract

Drought stress is a major abiotic factor limiting maize yield and stability. Although Jumonji C (JMJ) histone demethylases are known to regulate plant growth, development, and stress responses, their systematic characterization in maize has remained limited. Here, 27 *ZmJMJ* genes were identified in the maize genome through BLAST and conserved-domain analyses and classified into five subfamilies: JMJD6, KDM3/JHDM2, KDM4/JHDM3, KDM5/JARID1, and JmjC domain-only. Members within the same subfamily showed similar physicochemical properties, domain composition, and motif distribution, whereas clear divergence was observed among subfamilies. Chromosomal mapping revealed that *ZmJMJ* genes were unevenly distributed across nine chromosomes, with two interchromosomal homologous gene pairs, suggesting roles for segmental and/or whole-genome duplication in family expansion. Promoter analysis indicated widespread enrichment of elements related to light responsiveness, growth and development, and hormone and stress responses. Expression profiling showed that most *ZmJMJ* genes were highly expressed in leaves, while several displayed tissue specificity. Under drought stress, *ZmJMJ17a*, *ZmJMJ17b*, *ZmJMJ28*, and *ZmJMJ32* were significantly induced, highlighting them as promising candidates for functional studies and molecular breeding for drought tolerance in maize. This study provides a foundation for elucidating the evolution and functions of the *ZmJMJ* family and identifies candidate genes for drought-related functional validation and molecular breeding.

## Simple Summary

Maize is one of the world’s most important food crops, but its growth and yield are strongly affected by drought. Understanding how maize responds to water shortage is therefore important for food production and crop improvement. In this study, we examined a group of maize genes that may help control how plants turn other genes on or off during growth and stress. Using the latest maize genome information, we identified 27 members of this gene family and analyzed their characteristics, evolutionary relationships, and activity in different tissues. We also investigated how these genes respond to drought-like conditions. Several genes showed clear changes in activity under stress, and four genes were especially strongly induced. These results suggest that these genes may play important roles in how maize responds to drought. Our study provides updated information on this gene family in maize and offers useful candidate genes for future research. In the long term, this work may support the development of maize varieties with improved drought tolerance and help promote more stable crop production under changing environmental conditions.

## 1. Introduction

In eukaryotes, approximately 147 bp of DNA is wrapped around a histone octamer composed of two molecules each of H2A, H2B, H3, and H4, thereby forming a nucleosome [1]. Beyond the conserved globular core domains, histones are characterized by flexible, positively charged amino-terminal (N-terminal) tails, which serve as the principal targets of a wide range of post-translational modifications [2]. Previous studies have shown that histone tails can undergo multiple covalent modifications, including methylation, acetylation, phosphorylation, ADP-ribosylation, and ubiquitination. These modifications have been shown to influence the expression and activity of downstream proteins, alter cellular states, and affect embryogenesis and development, thereby serving as major carriers of epigenetic information and key regulators of biological processes [3]. Among these modifications, histone methylation and its dynamic, reversible demethylation have been recognized as particularly important for the precise regulation of gene expression and have therefore emerged as a central focus of research in plant epigenetics in recent years.

Histone methylation homeostasis is dynamically regulated by two antagonistic enzyme families: histone methyltransferases (HMTs) and histone demethylases (HDMs). Through their catalytic activities, methyl groups are deposited onto or removed from specific histone residues by these enzymes, thereby controlling the accessibility of genomic DNA to transcription factors [4,5]. In plants, this dynamic regulatory system is considered particularly critical for maintaining both the stability and plasticity of gene expression networks and for mediating developmental phase transitions, organogenesis, and responses to environmental cues. Of particular note, Jumonji C (JmjC) domain-containing proteins within the HDM family use Fe^2+^ and α-ketoglutarate as cofactors to specifically recognize and catalyze the demethylation of three distinct methylation states of histone lysine residues [6,7].

JmjC domain-containing proteins in plants have been shown to play critical roles in epigenetic regulation, plant growth, and development [8]. Across diverse plant species, members of the JMJ gene family have been systematically identified and functionally characterized. In *Arabidopsis thaliana*, for example, 21 JMJ proteins have been identified and classified into five subfamilies: KDM5/JARID1, KDM4/JHDM3, KDM3/JHDM2, JMJD6, and the JmjC domain-only subfamily (Yujiang Shi, 2004 [9]). Members of these subfamilies have been shown to fulfill distinct roles in chromatin demethylation and related physiological processes. Within the JARID1/KDM5 subfamily, *AtJMJ14* encodes an H3K4 demethylase capable of removing H3K4me1, H3K4me2, and H3K4me3, thereby contributing to flowering-time regulation [7]. Other members of the same subfamily, *AtJMJ16* and *AtJMJ17*, have been implicated in leaf senescence and osmotic stress responses, respectively [10,11]. In addition, within the KDM4/JHDM3 subfamily, *AtJMJ11/ELF6* and *AtJMJ12/REF6* have been shown to interact with BES1, a transcription factor in the brassinosteroid (BR) signaling pathway [12]. These findings suggest that such demethylases may be precisely recruited to target loci via interactions with sequence-specific transcription factors, thereby enabling locus-specific chromatin demethylation. Beyond *Arabidopsis*, studies in crop species have further underscored the importance of the JMJ family in stress responses and fruit ripening. In upland cotton, *GhJMJ34* and *GhJMJ40* have been shown to markedly enhance tolerance to salt and osmotic stresses [13]. In tomato, *SlJMJ6* promotes fruit ripening by removing H3K27me3 and activating the key ripening genes *ACS4* and *ACO1*, whereas *SlJMJ7* regulates ripening by demethylating H3K4me1/2/3 and thereby repressing *DML2* expression [14,15]. In addition, *SlJMJC3* specifically removes repressive histone marks at *HSP* loci, including H3K9me1/3 and H3K27me3, thereby enhancing thermotolerance [16]. In rice, *DT2*, a JmjC domain-containing gene encoding an H3K9me2 demethylase, has been shown to regulate drought tolerance [17].

Maize (*Zea mays* L.) is one of the world’s most important staple crops and also serves as a major source of animal feed and industrial raw materials. Its growth and development are highly sensitive to water availability. Drought has been shown to substantially reduce maize yields; data from the United States spanning 1961 to 2021 indicate that drought can cause yield losses of 17–29%, thereby posing a substantial threat to food security [18]. Previous studies have provided important insights into the maize JmjC/JMJ histone demethylase family [19]; however, an updated analysis based on the latest reference genome, together with drought-focused expression profiling, remains lacking. In this study, we conducted a genome-wide re-evaluation of the maize JMJ gene family using the Zm-B73-REFERENCE-NAM-5.0 genome assembly. Through phylogenetic, structural, cis-element, and expression analyses, including qRT-PCR under PEG-simulated drought stress, we identified drought-responsive candidate genes and provided an updated foundation for future functional studies of epigenetic regulation in maize drought response.

## 2. Materials and Methods

### 2.1. Identification of ZmJMJ Gene Family Members

The maize genome assembly (version Zm-B73-REFERENCE-NAM-5.0), GFF annotation file, and protein sequence files were retrieved from the Ensembl Plants database (https://plants.ensembl.org/index.html) (accessed on 5 December 2025). The hidden Markov model (HMM) profile of the JmjC domain (PF02373) was downloaded from InterPro (https://www.ebi.ac.uk/interpro/) (accessed on 6 December 2025) and used as a query to search the maize protein dataset using HMMER. Candidate members of the maize *ZmJMJ* gene family were initially identified using an E-value threshold of 1 × 10^−5^. The protein sequences of the candidate genes were then subjected to domain validation using the Conserved Domain Database (CDD) (https://www.ncbi.nlm.nih.gov/Structure/bwrpsb/bwrpsb.cgi) (accessed on 6 December 2025), and only proteins containing the JmjC domain were retained. The physicochemical properties of the ZmJMJ proteins, including amino acid length, molecular weight, isoelectric point (pI), grand average of hydropathicity, and aliphatic index, were calculated using the Protein Parameter Calculator plugin in TBtools-II v2.331.

### 2.2. Chromosomal Localization and Synteny Analysis of the ZmJMJ Gene Family

Using the maize GFF3 annotation file, the chromosomal position information and density data of the *ZmJMJ* gene family members were extracted with TBtools-II v2.331, and the chromosomal distribution of the *ZmJMJ* genes was visualized using the Gene Location plugin. In addition, all *ZmJMJ* genes were assigned standardized nomenclature on the basis of their homology to *AtJMJ* members.

Genome sequence files and GFF3 annotation files for *Arabidopsis thaliana*, *Populus trichocarpa*, *Zea mays*, and *Oryza sativa* were downloaded for comparative analysis. Syntenic relationships among *ZmJMJ* genes and between *ZmJMJ* genes and their counterparts in other species were analyzed using the MCScanX plugin in TBtools-II v2.331 and subsequently visualized using TBtools-II v2.331.

### 2.3. Gene Structure and Conserved Domain Analysis of the ZmJMJ Gene Family

Using the maize GFF3 annotation file, the gene structures of *ZmJMJ* members were generated and visualized with the Visualize Gene Structure plugin in TBtools-II v2.331. Conserved motifs in *ZmJMJ* protein sequences were identified using the MEME online server (http://meme.nbcr.net/meme/intro.html) (accessed on 6 January 2026), with the motif width range set to 6–15 amino acids.

### 2.4. Construction of the Phylogenetic Tree for the ZmJMJ Gene Family

JMJ protein sequences from *Arabidopsis thaliana*, *Oryza sativa*, *Triticum aestivum*, *Vitis vinifera*, and *Zea mays* were subjected to multiple sequence alignment using MEGA 11. A phylogenetic tree was then constructed using the neighbor-joining (NJ) method with 1000 bootstrap replicates. Protein sequences from *Arabidopsis thaliana*, rice, wheat, and grape were downloaded from the UniProt database (https://www.uniprot.org/) (accessed on 16 January 2026). The resulting phylogenetic tree was exported in Newick format and subsequently visualized using ChiPlot (https://www.chiplot.online/) (accessed on 18 January 2026).

### 2.5. Cis-Regulatory Element Analysis of the Promoter Regions of ZmJMJ Genes

The 2000-bp upstream sequences from the translation start site (ATG) of maize *ZmJMJ* genes were extracted using TBtools-II. Cis-regulatory elements were predicted using the PlantCARE database (http://bioinformatics.psb.ugent.be/webtools/plantcare/html/) (accessed on 27 January 2026) and then visualized with the ggplot2, tidyverse, readxl, ggh4x, ggtree, tidytree, treeio, ggfun, ape, phytools, aplot, patchwork, and writexl package in R 4.4.2.

### 2.6. Protein–Protein Interaction Network Analysis of ZmJMJ Proteins

All ZmJMJ protein sequences were submitted to the STRING database (https://cn.string-db.org/) (accessed on 3 February 2026), with *Arabidopsis thaliana* homologs used as references. Proteins without predicted interaction partners were removed, and the resulting TSV file was downloaded. The protein–protein interaction (PPI) network was then visualized using Cytoscape 3.10.3.

### 2.7. Expression Pattern Analysis of the ZmJMJ Gene Family

Using *ZmJMJ* gene IDs as queries, FPKM expression data across different tissue types were retrieved from the public ZEAMAP database (https://db.cngb.org/zeamap/module/transcriptomics/single_expression_viewer) (accessed on 5 February 2026). The data were then log2-transformed and visualized as a heatmap using TBtools-II v2.331. The tissues analyzed included the vegetative meristem, embryo, germinating kernels, endosperm, mature leaf, root meristem zone, leaf zone 1, leaf zone 2, leaf zone 3, endosperm crown, root cortex, primary root, secondary root, root elongation zone, and mature pollen.

### 2.8. Plant Materials and Drought Treatment

The maize hybrid ‘Hongshuo 822’, which is widely cultivated locally and exhibits stable growth performance, was selected as the experimental material. Fully mature and well-filled seeds were sown in seedling pots and grown in a growth chamber. The growth conditions were maintained at 25 °C, with a relative humidity of approximately 70%, a light intensity of 450 μmol m^−2^ s^−1^, and a photoperiod of 16 h light/8 h darkness. When the seedlings had reached the three-leaf stage, individuals with uniform growth were selected and transferred to a 15% (*w*/*v*) PEG6000 (Guangda Hengyi Technology Co., Ltd., Beijing, China) solution to simulate drought stress. Using the 0 h time point as the control, leaf samples were collected at 3, 6, 12, and 24 h after PEG treatment. Three independent biological replicates were included for each treatment, and each biological replicate consisted of three seedlings. Following collection, all samples were immediately frozen in liquid nitrogen and stored at −80 °C until further analysis.

### 2.9. qRT–PCR Analysis

Total RNA was extracted from maize leaves using the RNAprep Pure Plant Kit (TIANGEN, Beijing, China) according to the manufacturer’s instructions. Total RNA was reverse-transcribed into complementary DNA (cDNA) using the PrimeScript RT Reagent Kit (Perfect Real Time; TaKaRa, Beijing, China) and the HiScript^®^ II 1st Strand cDNA Synthesis Kit (Vazyme, Nanjing, China). Real-time quantitative PCR was subsequently performed using the SYBR Green Premix Pro Taq HS qPCR Kit III (Accurate Biotechnology [Hunan] Co., Ltd., Changsha, China) on a Gentier 96E real-time PCR system (Tianlong Technology Co., Ltd., Xi’an, China). Three technical replicates were performed for each cDNA sample, and relative gene expression levels were calculated using the 2^−ΔΔCt^ method [20]. The primers used in this study (Table S1) were synthesized by Bioengineering Co., Ltd. (Shanghai, China).

### 2.10. Data Analysis

Data were processed using Microsoft Excel. Statistical analyses were performed using SPSS 22.0 software. Group differences were assessed by one-way ANOVA followed by Dunnett’s multiple comparisons test. Figures were prepared using GraphPad Prism 8, R, and Adobe Illustrator 2023.

## 3. Results

### 3.1. Identification and Physicochemical Characterization of the Maize JMJ Gene Family

By integrating domain validation with NCBI BLAST web server and manually removing redundant sequences, a total of 27 *JMJ* genes were identified in maize. To comprehensively characterize the structural and physicochemical features of the *ZmJMJ* gene family, analyses were conducted on amino acid length, molecular weight (MW), theoretical isoelectric point (pI), instability index (II), grand average of hydropathicity (GRAVY), and aliphatic index (AI) (Table 1). The results showed that the *ZmJMJ* proteins ranged from 118 to 1875 amino acids in length, with an average of approximately 786 aa, and in molecular weight from 13.04 to 212.12 kDa, with a mean of approximately 88.18 kDa. Among them, *ZmJMJ18* was identified as the largest protein (1875 aa; 212.12 kDa), whereas *ZmJMJ34* and *ZmJMJ33* were the smallest proteins (118–127 aa; 13–14 kDa). The instability index of all family members exceeded 40 (41.12–67.96; mean, approximately 53.73), indicating that they were predicted to be unstable proteins. The aliphatic index ranged from 52.89 to 91.93, with an average of approximately 73.66, suggesting that most members exhibited moderate to high aliphaticity. All GRAVY values were negative (−0.115 to −0.968; mean, approximately −0.506), indicating that the family proteins were predominantly hydrophilic and likely existed in soluble form.

### 3.2. Chromosomal Localization and Synteny Analysis of the ZmJMJ Family

**Figure 1:**
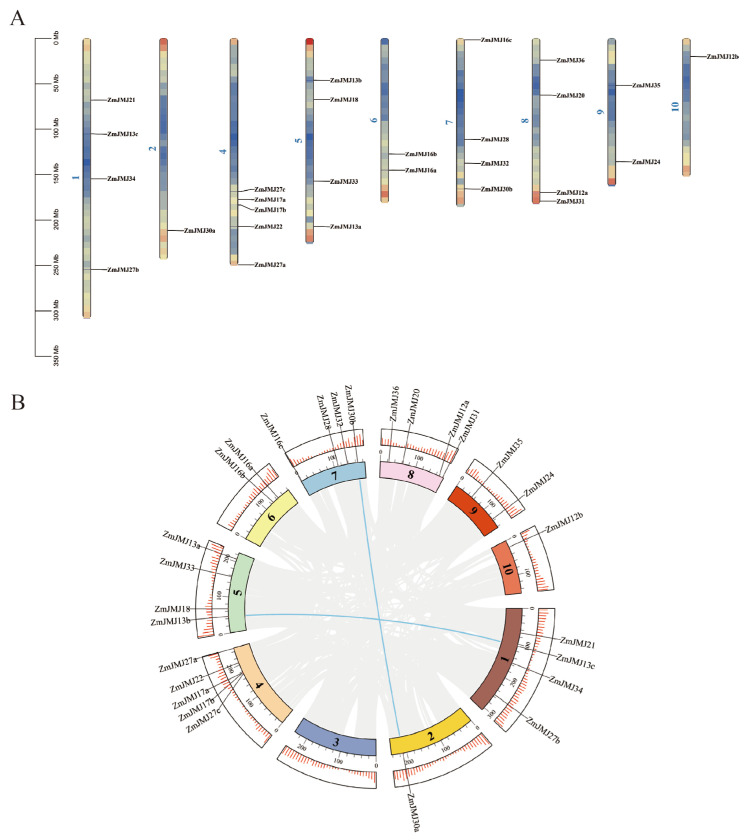
Chromosomal localization and synteny analysis of the *ZmJMJ* gene family. (**A**) Chromosomal distribution of *ZmJMJ* genes in maize. The vertical bars represent chromosomes, and the numbers (1–10) indicate chromosome identities. The scale on the left represents chromosome length (Mb). (**B**) Synteny analysis of the *ZmJMJ* gene family in maize. Blue lines within the circle indicate segmentally duplicated gene pairs of *ZmJMJ* genes The colored segments represent different chromosomes, the numbers (1–10) indicate chromosome identities and the red tick marks represent gene density.

To elucidate the chromosomal distribution patterns and duplication-associated expansion of the *ZmJMJ* gene family in the maize genome, chromosomal localization and synteny analyses were performed for all 27 *ZmJMJ* genes. Chromosomal mapping showed that *ZmJMJ* members were unevenly distributed across nine chromosomes (Chr1, Chr2, Chr4, Chr5, Chr6, Chr7, Chr8, Chr9, and Chr10), whereas no member was detected on Chr3 (Figure 1A). Among these chromosomes, the highest enrichment was observed on Chr4, where five genes were mapped. Four genes were located on each of Chr1, Chr5, Chr7, and Chr8; two genes were found on each of Chr6 and Chr9; and only one gene was identified on each of Chr2 and Chr10. Several genes also exhibited adjacent or locally clustered distributions on the same chromosome, as exemplified by *ZmJMJ16a/16b* on Chr6 and gene clusters on Chr5 and Chr8. Intraspecific synteny analysis further revealed two homologous gene pairs distributed across four chromosomes, namely *ZmJMJ13b/ZmJMJ13c* and *ZmJMJ30a/ZmJMJ30b* (Figure 1B), suggesting that the expansion of this family was primarily associated with segmental duplication rather than with local tandem duplication alone.

### 3.3. Phylogenetic and Evolutionary Analysis of the ZmJMJ Gene Family

**Figure 2:**
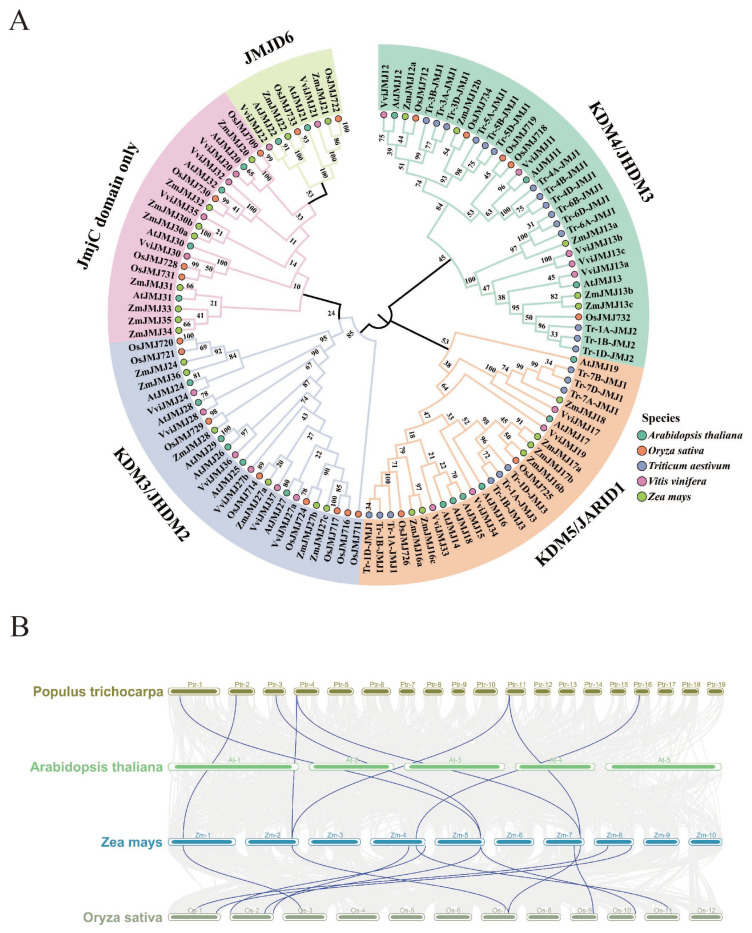
Phylogenetic and evolutionary analysis of *ZmJMJ* gene family members. (**A**) Phylogenetic tree of JMJ proteins from maize, grape, rice, *Arabidopsis thaliana*, and wheat. Different background colors indicate different JMJ subfamilies. Colored circles beside gene names represent species identity. Numbers at the nodes indicate bootstrap support values. The numbers at the nodes represent bootstrap support values, and the numbers in the inner circle indicate homology values. (**B**) Syntenic relationships between *ZmJMJ* genes and their counterparts in *Arabidopsis thaliana*, rice, and *Populus trichocarpa*. Different colored horizontal bars represent chromosomes from different species, and the labels on the bars indicate chromosome identities. Blue lines indicate syntenic gene pairs involving JMJ genes, while gray lines represent background collinear blocks in the compared genomes.

To elucidate the phylogenetic relationships between the *ZmJMJ* family and JMJ proteins from other plant species, as well as their potential functional diversification, a phylogenetic tree was constructed using JMJ protein sequences from *Arabidopsis thaliana*, *Oryza sativa*, *Triticum aestivum*, *Vitis vinifera*, and maize (Figure 2A). The results showed that the *ZmJMJ* family could be divided into five subfamilies: KDM5/JARID1, KDM4/JHDM3, KDM3/JHDM2, JMJD6, and the JmjC domain-only subfamily. *ZmJMJ12a/12b* and *ZmJMJ13a/13b/13c* were clustered within the KDM4/JHDM3 subfamily; *ZmJMJ16a/16c*, *ZmJMJ17a/17b*, and *ZmJMJ18* were assigned to KDM5/JARID1; *ZmJMJ24*, *ZmJMJ28*, *ZmJMJ27a/27b/27c*, and *ZmJMJ36* were grouped into KDM3/JHDM2; *ZmJMJ21* and *ZmJMJ22* were classified into JMJD6; whereas *ZmJMJ20*, *ZmJMJ30a/30b*, *ZmJMJ31*, *ZmJMJ33*, *ZmJMJ34*, and *ZmJMJ35* were clustered within the JmjC domain-only subfamily.

To investigate the evolutionary conservation of the *ZmJMJ* family across species, a multispecies synteny analysis was performed. The results showed that syntenic relationships between maize and rice were the most abundant (Figure 2B) and were markedly stronger than those observed between maize and *Arabidopsis thaliana* or *Populus trichocarpa*. These findings suggested that *ZmJMJ* genes were more highly conserved within the Poaceae, whereas more pronounced divergence had occurred following the divergence of monocotyledonous and dicotyledonous lineages. In addition, several maize genes corresponded to single homologous syntenic blocks in other species, suggesting that duplicated copies had been retained in the maize lineage and may have undergone functional diversification.

### 3.4. Motif Composition, Conserved Domains, and Gene Structure Analysis of the ZmJMJ Gene Family

**Figure 3:**
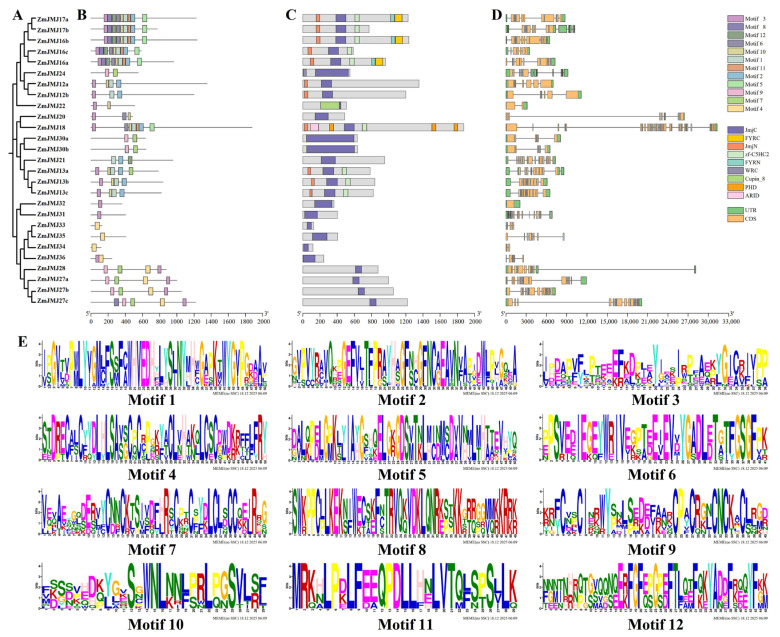
Motif composition, conserved domain architecture, and gene structure analysis of maize JMJ genes. (**A**) Phylogenetic tree of the maize JMJ gene family. (**B**) Conserved motifs. Different colors represent different conserved motifs. (**C**) Domain architecture. Different colors represent different conserved domains. (**D**) Gene structure. Orange boxes indicate UTRs, green boxes indicate CDS regions, and black lines represent introns. (**E**) Conserved sequence logos. The letters represent amino acid residues in each conserved motif; the height of each letter indicates the relative conservation at that position, and different colors denote different residue types according to their physicochemical properties.

To further characterize the structural features and functional conservation of the maize *JMJ* gene family, a comprehensive analysis was conducted on the motif composition, functional domain organization, and gene structures of 27 *ZmJMJ* genes. The results showed that the 27 *ZmJMJ* members could be divided into several stable clades, within which multiple groups of closely related genes were identified, including *ZmJMJ17a/17b*, *ZmJMJ12a/12b*, *ZmJMJ30a/30b*, and *ZmJMJ27a/27b/27c* (Figure 3A), suggesting that gene duplication contributed to family expansion. Within the same clade, the types and arrangement of conserved motifs were found to be highly similar, and the motif composition of closely related gene pairs was nearly identical, whereas a few members exhibited fewer motifs (Figure 3B), possibly reflecting structural simplification. Domain analysis further showed that most proteins contained the core JmjC domain characteristic of the *JMJ* family and were accompanied, in different clades, by distinct combinations of JmjN, FYRN/FYRC, PHD, and ARID domains (Figure 3C), indicating modular domain organization and potential functional diversification. With respect to gene structure, exon–intron organization varied substantially among members, whereas genes within the same clade displayed more similar structural organization (Figure 3D). Together with the conservation of key residues revealed by motif sequence logos (Figure 3E), these findings indicate a strong overall concordance between the phylogenetic classification of the *ZmJMJ* family and the corresponding protein domain architecture, motif composition, and gene structure.

### 3.5. Cis-Regulatory Element Analysis of the Upstream Promoter Regions of the ZmJMJ Genes

**Figure 4:**
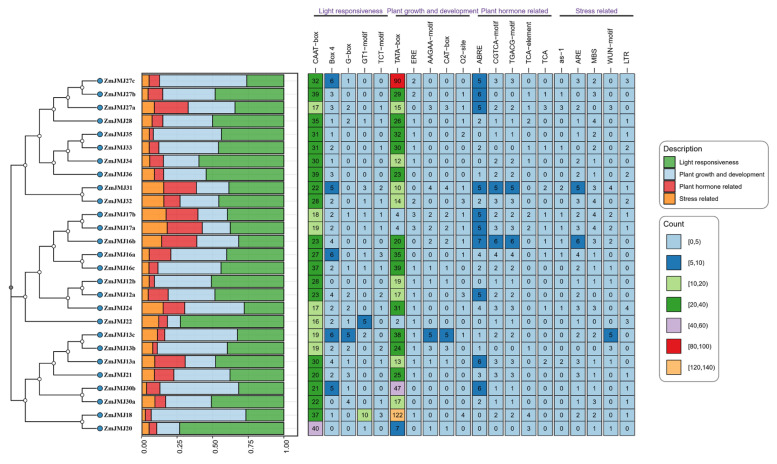
Cis-acting element analysis of the promoter regions of maize *JMJ* genes. The left vertical axis lists the 27 *ZmJMJ* gene identifiers. The central stacked bar chart shows the total number of cis-acting elements classified by functional categories. The heatmap on the right presents representative cis-acting elements along the horizontal axis, with the frequency of occurrence indicated numerically within each cell.

To further investigate the potential regulatory mechanisms of *ZmJMJ* genes, cis-regulatory elements were predicted within the promoter regions (2000 bp upstream of the translation start site) of 27 *ZmJMJ* genes (Figure 4). The results showed that *ZmJMJ* promoters were generally enriched in core transcriptional elements and elements associated with growth, development, and light responsiveness, including the TATA-box, CAAT-box, Box 4, and G-box. These findings suggested that the family shares a relatively conserved basal transcriptional regulatory framework and may be broadly involved in growth, developmental, and light-signaling processes. At the same time, marked differences were observed among individual members in the abundance and combination of hormone- and stress-related cis-elements, such as ABRE, CGTCA/TGACG, ARE, MBS, and LTR, indicating that promoter-mediated regulation may have diverged during gene duplication and evolutionary diversification. Of particular note, significant enrichment of TATA-box elements was detected in *ZmJMJ18* and *ZmJMJ27c*, implying potentially strong transcriptional activity. In *ZmJMJ16b* and *ZmJMJ31*, ABRE and jasmonate-related elements (CGTCA/TGACG), together with stress-responsive elements such as ARE and MBS, were more prominently represented, suggesting a greater likelihood of involvement in hormone-mediated stress responses. By contrast, MBS, a drought-related cis-element, was relatively enriched in *ZmJMJ28*, *ZmJMJ32*, *ZmJMJ17a*, and *ZmJMJ17b*. Collectively, these results indicate that, against a conserved regulatory background associated with development and light responsiveness, the *ZmJMJ* family exhibits pronounced member-specific differences in hormone- and stress-related regulatory potential.

### 3.6. Spatiotemporal Expression Patterns and Protein–Protein Interaction Network Analysis of the ZmJMJ Genes

**Figure 5:**
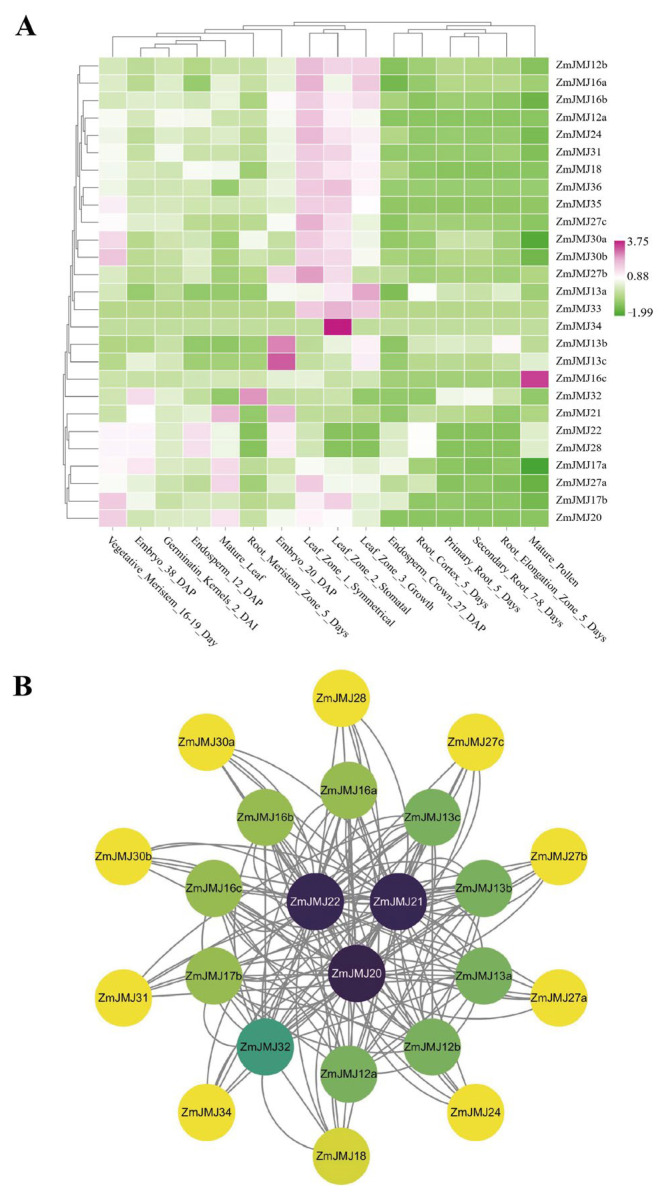
Spatiotemporal expression pattern analysis and protein–protein interaction network of *ZmJMJ* genes. (**A**) Hierarchical clustering of gene expression profiles across different maize tissues and developmental stages. Relative expression levels (center) are indicated according to the color scale shown on the right, with green and pink representing low and high expression abundance, respectively. Gene names are displayed on the right, and tissue types and developmental stages are shown at the bottom. (**B**) Network analysis of *ZmJMJ* genes. Each node represents a *ZmJMJ* gene, and the connecting lines indicate associations between gene pairs. Node colors represent the relative connectivity of each gene in the network, with darker colors indicating higher connectivity.

To systematically characterize the spatiotemporal expression patterns of the *ZmJMJ* gene family across different maize tissues and developmental stages and to identify key candidate genes, expression profiles of *ZmJMJ* genes were generated using publicly available transcriptomic data (Figure 5A). The results showed that the *ZmJMJ* family exhibited pronounced tissue-specific expression patterns. Most *ZmJMJ* genes were highly expressed in leaf-related samples but showed relatively low expression in most root tissues and mature pollen. For example, *ZmJMJ34* displayed markedly high, stomata-specific expression, whereas *ZmJMJ21* was highly expressed in mature leaves. By contrast, *ZmJMJ16c* showed pollen-specific high expression, with comparatively low expression in other tissues. In addition, *ZmJMJ13b* and *ZmJMJ13c* exhibited pronounced stage-specific high expression during embryonic development at approximately 20 days after pollination. Homologous gene pairs, such as *ZmJMJ30a/30b*, displayed similar expression trends, suggesting a degree of functional redundancy.

To evaluate the potential functional associations and coordinated relationships among *ZmJMJ* family members, an interaction network of *ZmJMJ* genes was constructed (Figure 5B). The results showed that *ZmJMJ20*, *ZmJMJ21*, and *ZmJMJ22* occupied central positions in the network and exhibited the highest connectivity, suggesting that they may serve as hub genes with central regulatory roles within the family.

### 3.7. Expression Patterns of the ZmJMJ Gene Family Under Drought Stress

**Figure 6:**
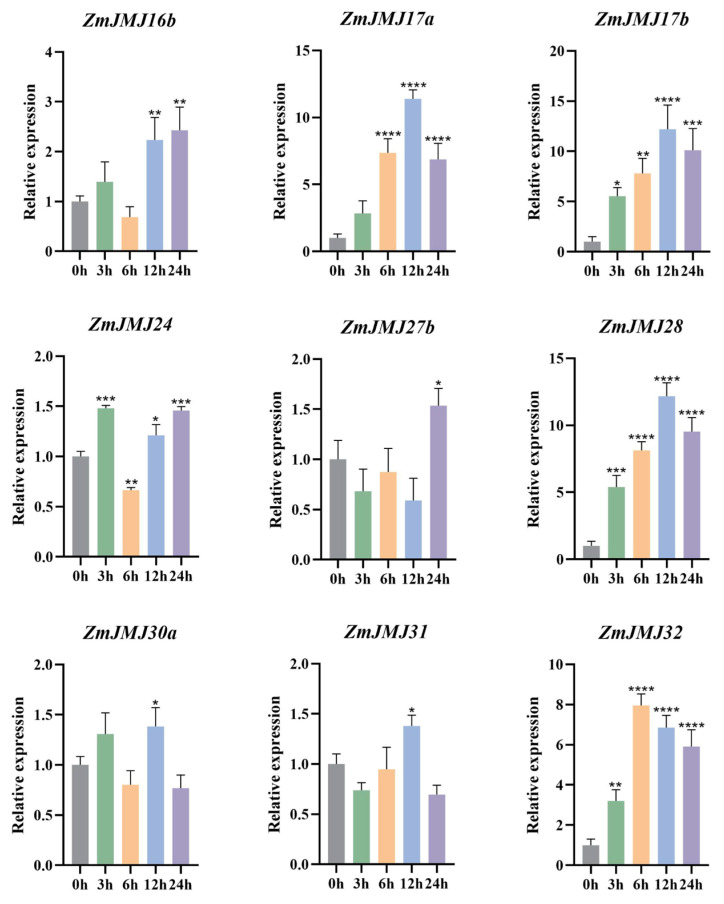
Expression pattern analysis of maize *ZmJMJ* genes under drought stress. Error bars represent the standard deviation of three biological replicates. Asterisks above the bars indicate significant differences in gene expression levels relative to the control (0 h): * *p* < 0.05; ** *p* < 0.01; *** *p* < 0.001; **** *p* < 0.0001.

On the basis of promoter cis-element prediction and tissue expression profiling, nine *ZmJMJ* genes were selected for further analysis, and their transcriptional responses to drought stress were examined by qRT–PCR (Figure 6). The results showed that these genes exhibited distinct temporal expression patterns at 0, 3, 6, 12, and 24 h of drought treatment. Among them, *ZmJMJ17a*, *ZmJMJ17b*, *ZmJMJ28*, and *ZmJMJ32* were significantly induced and were identified as the primary drought-responsive genes. Peak expression of *ZmJMJ17a/17b* and *ZmJMJ28* was observed at 12 h, and relatively high transcript levels were maintained at 24 h, whereas *ZmJMJ32* was rapidly upregulated at 6 h and remained highly expressed thereafter. By contrast, *ZmJMJ16b* was upregulated during the 12–24 h interval, and *ZmJMJ27b* was significantly induced mainly at 24 h, suggesting that these genes may participate in adaptive regulation during the intermediate and late stages of stress. In comparison, only limited expression changes were detected for *ZmJMJ30a* and *ZmJMJ31*, with slight upregulation observed only at 12 h. Collectively, *ZmJMJ17a*, *ZmJMJ17b*, *ZmJMJ28*, and *ZmJMJ32* may be regarded as candidate genes for subsequent functional validation of drought tolerance.

## 4. Discussion

Maize is one of the world’s most important cereal crops and serves as a major source of food, feed, and industrial raw materials. Its yield and quality are strongly influenced by water availability [21]. During plant responses to drought stress, epigenetic regulation can enable the rapid, reversible, and finely tuned modulation of stress-responsive genes through alterations in chromatin state and transcriptional reprogramming, thereby enhancing adaptation and stress tolerance [4]. Among the regulators involved, the JMJ protein family is recognized as a major class of histone demethylases [22]. By removing methylation marks from specific histone sites, members of this family can alter transcriptional activity and thereby participate in key biological processes, including growth and development, hormone signaling, and stress responses. To date, the *JMJ* gene family has been identified and functionally characterized in multiple plant species, including *Arabidopsis thaliana* [7], upland cotton [13], *Vitis vinifera* L. [23], Rosa chinensis [24], and Chinese cabbage [25].

In Qian et al. [19], 19 members of the *JMJ* gene family were identified in maize using the V3.0 reference genome, and their phylogenetic relationships, gene structures, and expression patterns were systematically characterized; qRT–PCR analysis further showed that some of these genes responded to heat stress. Building on that work, the maize *JMJ* gene family was re-evaluated in the present study using the V5.0 reference genome, and 27 family members were identified, representing eight additional members relative to the previous report. This discrepancy was likely attributable to improvements in the reference genome assembly, refinement of gene annotation, and differences in the criteria used for family identification. Compared with previous studies, the present work further focused on expression changes in *ZmJMJ* genes under PEG-simulated drought conditions and identified drought-inducible candidate genes, thereby providing an updated resource for dissecting the epigenetic regulatory mechanisms underlying drought responses in maize.

Previous studies have also shown that the *JMJ* gene family varies markedly in size among plant species. For example, 21 *JMJ* members have been identified in *Arabidopsis thaliana* [7], 20 in rice [26], 24 in wheat [27], 23 in tomato [28], and as many as 48 in soybean [29], which is the highest number reported among these species. Phylogenetic analysis further showed that the ZmJMJ proteins could be classified into five subfamilies—JMJD6, KDM3/JHDM2, KDM4/JHDM3, KDM5/JARID1, and the JmjC domain-only subfamily—consistent with findings reported for *Arabidopsis thaliana*, soybean, rice, Chinese cabbage, and tomato [25]. These results suggest that diversification of the *ZmJMJ* gene family occurred relatively early and that this family has remained evolutionarily conserved across plant lineages. In addition, the 27 *ZmJMJ* genes were found to be distributed across nine maize chromosomes, and two homologous gene pairs (*ZmJMJ13b/ZmJMJ13c* and *ZmJMJ30a/ZmJMJ30b*) were identified on four different chromosomes, indicating that the expansion of the *ZmJMJ* family in maize was driven primarily by whole-genome duplication and segmental duplication events.

Gene structure analysis revealed a consistent pattern across the *ZmJMJ* family at the levels of gene organization, motif composition, and domain architecture. Marked differences in exon number and structural organization were observed among different subfamilies, whereas the exon structures of most members within the same subfamily remained relatively conserved, suggesting that strong structural constraints were maintained following family diversification. This conclusion was further supported by motif analysis, which showed that each subfamily possessed characteristic motif combinations and arrangements, whereas greater similarity was retained among members within the same subfamily. These findings indicate that the primary patterns of evolutionary divergence and functional specialization occurred at the subfamily level. Domain analysis yielded a similar pattern, with clear differences in domain type and number among subfamilies, but stable combinations of conserved domains were retained within each subfamily. In particular, the JmjC domain-only subfamily exhibited a more simplified architecture, consistent with the shorter protein sequences identified in this family, suggesting that domain loss and/or rearrangement may have occurred and may have contributed to functional diversification [23]. Of particular note, an additional ARID domain was identified in *ZmJMJ18*, implying that this protein may have acquired novel functional properties.

Transcriptional regulatory elements within promoter regions are essential for transcription initiation and gene expression regulation [30]. In the present study, promoter regions of the *ZmJMJ* genes were found to contain predominantly light-responsive elements, growth- and development-related elements, as well as hormone- and stress-responsive elements, consistent with previous reports [31,32]. These findings suggest that the *ZmJMJ* family may participate not only in basal developmental regulation but also in responses to environmental changes and endogenous signaling cues. Of particular note, large numbers of CAAT-box and TATA-box elements were identified in the promoters of all *ZmJMJ* genes, indicating that this family possesses a conserved core promoter architecture and a relatively well-established basal transcriptional regulatory framework, which may provide the basis for further fine-tuned regulation under different tissue-specific and environmental conditions [19]. In addition, ABRE (ABA-responsive element) and MBS (drought-related element) were enriched in a subset of members, suggesting that these genes may be preferentially regulated by drought signals mediated through the ABA–MYB pathway. Jasmonate-related elements (CGTCA/TGACG) and salicylic acid–related elements (TCA/TCA-element) were also found to be differentially distributed among individual genes, indicating that *ZmJMJ* genes may operate within a multilayered hormone signaling network and contribute to the balance between stress responses and growth and development through the modulation of histone methylation status.

Previous studies have shown that *JMJ* genes in wheat are most highly expressed in roots and spikes [27], whereas *BpJMJ* genes are predominantly expressed in roots and flowers [32]. By contrast, the present study showed that most *ZmJMJ* genes were highly expressed in leaf-related samples but exhibited little or no expression in roots, suggesting that the *ZmJMJ* family may be broadly involved in chromatin regulatory processes associated with leaf development, mesophyll differentiation, and photosynthesis-related activities during vegetative growth. This interpretation is further supported by the enrichment of light-responsive elements identified in the promoter regions of these genes. In addition, *ZmJMJ34* was found to be markedly enriched in stomatal tissues, implying a potential role in stomatal development and/or the regulation of stomatal opening and closure. *ZmJMJ16c* was specifically expressed in mature pollen, suggesting an association with male gametophyte development, pollen maturation, or pollen tube growth [33]. Moreover, the marked upregulation of *ZmJMJ13b/13c* in embryos at approximately 20 d suggested that these genes may participate in epigenetic regulation during embryogenesis and embryo maturation [34].

Dynamic changes in gene expression under drought stress are typically regulated by histone post-translational modifications and DNA methylation in plants. Previous studies have shown that *AtJMJ27* positively regulates drought tolerance in *Arabidopsis thaliana* [35], whereas *OsDT2/JMJ720* and *OsJMJ710* act as negative regulators of drought tolerance in rice [17,36]. By contrast, the functions of *JMJ* family genes in drought responses in maize have not yet been systematically investigated. In this study, qRT–PCR analysis showed that the nine candidate genes exhibited distinct induction intensities and temporal patterns over the 0–24 h drought treatment period, suggesting that *ZmJMJ*-mediated epigenetic regulation may participate in multiple phases of the drought response. Of particular note, *ZmJMJ17a*, *ZmJMJ17b*, *ZmJMJ28*, and *ZmJMJ32* were markedly upregulated under drought conditions and displayed strong induction levels. Peak expression of *ZmJMJ17a/17b* and *ZmJMJ28* was reached at 12 h and remained at relatively high levels at 24 h, whereas *ZmJMJ32* was rapidly induced at 6 h and subsequently maintained high expression. These expression patterns suggest that these genes may occupy key positions in drought signal transduction and transcriptional reprogramming and may participate in the regulation of stress-responsive gene expression or chromatin remodeling [37]. In addition, promoter cis-element analysis showed that the promoters of these four drought-responsive candidate genes each harbored four MBS elements. This finding was broadly consistent with the drought-inducible expression patterns revealed by qRT–PCR, suggesting that these genes may participate in a MYB-mediated regulatory network governing drought responses. The PPI network further showed that several drought-responsive candidate genes were incorporated into the interaction network. Among them, *ZmJMJ32* was predicted to be associated with multiple core nodes, suggesting that it may function within a broader regulatory network. By contrast, *ZmJMJ28* was positioned at the periphery of the network, implying that its function may be relatively specialized and may depend on cooperative interactions with other JMJ proteins. In the future, functional validation of drought-inducible candidate genes such as *ZmJMJ17a*, *ZmJMJ17b*, *ZmJMJ28*, and *ZmJMJ32* could be further pursued in combination with gene-editing approaches to explore their potential utility in breeding drought-tolerant maize varieties.

## 5. Conclusions

In the present study, 27 *ZmJMJ* genes were identified in the maize genome through bioinformatic analyses and were classified into five subfamilies on the basis of their phylogenetic relationships with *Arabidopsis thaliana*. Members within the same subfamily were found to be largely consistent in their physicochemical properties, conserved domains, and motif composition, whereas pronounced differences were observed among subfamilies, reflecting a clear pattern of evolutionary diversification within this family. Promoter cis-element analysis showed that the promoters of *ZmJMJ* genes contain elements related to light responsiveness, growth and development, as well as hormone and stress responses, suggesting that these genes may participate in both developmental regulation and stress responses. Spatiotemporal expression profiling further revealed that most *ZmJMJ* genes were highly expressed in leaf tissues, implying potential roles in vegetative growth and leaf-related biological processes. Moreover, qRT–PCR analysis under drought stress demonstrated that *ZmJMJ17a*, *ZmJMJ17b*, *ZmJMJ28*, and *ZmJMJ32* were significantly induced during drought treatment, identifying them as candidate genes involved in drought tolerance regulation in maize. Collectively, these findings provide a theoretical foundation for subsequent functional characterization and molecular breeding efforts.

## Figures and Tables

**Table 1:** Physicochemical properties of the *ZmJMJ* genes.

Sequence Name	Sequence ID	Protein Length (aa)	Molecular Weight (kDa)	pI	Instability Index	Aliphatic Index	Grand Average of Hydropathicity
ZmJMJ12a	Zm00001eb365340	1353	149,329.8	9.05	50.22	68.72	−0.598
ZmJMJ12b	Zm00001eb409800	1199	133,714.3	8.86	47.5	65.05	−0.668
ZmJMJ13a	Zm00001eb251380	785	88,718.77	6.11	52.31	74.04	−0.335
ZmJMJ13b	Zm00001eb224940	839	93,913.5	8.45	60.05	71.36	−0.454
ZmJMJ13c	Zm00001eb025020	821	92,079.18	8.24	57.14	69.14	−0.489
ZmJMJ16a	Zm00001eb284570	963	108,903.5	6.74	53.9	68.93	−0.498
ZmJMJ16b	Zm00001eb280460	1235	138,597	6.38	59.51	74.69	−0.493
ZmJMJ16c	Zm00001eb298510	587	66,712.51	6.72	50.81	65.43	−0.54
ZmJMJ17a	Zm00001eb191790	1227	136,382.3	7.66	60.11	73.21	−0.464
ZmJMJ17b	Zm00001eb191820	771	86,877.58	6.73	58.58	72.23	−0.525
ZmJMJ18	Zm00001eb229050	1875	212,118.2	5.79	52.17	91.93	−0.25
ZmJMJ20	Zm00001eb342240	486	56,253.09	5.37	46.97	75.78	−0.388
ZmJMJ21	Zm00001eb018970	953	108,862	5.36	51.01	87.52	−0.31
ZmJMJ22	Zm00001eb200180	509	57,294.75	8.93	48.43	86.46	−0.258
ZmJMJ24	Zm00001eb394830	550	60,925.3	8.09	58.25	73.78	−0.39
ZmJMJ27a	Zm00001eb209780	999	111,903.5	7.59	44.94	68.11	−0.737
ZmJMJ27b	Zm00001eb049830	1055	119,440.5	8.95	53.56	63.2	−0.815
ZmJMJ27c	Zm00001eb190750	1219	136,361.4	7.94	56.06	61.86	−0.848
ZmJMJ28	Zm00001eb311870	877	98,681.92	6.71	55.46	68.95	−0.665
ZmJMJ30a	Zm00001eb106740	635	68,520.8	5.14	67.96	53.28	−0.968
ZmJMJ30b	Zm00001eb323940	640	69,267.59	5.23	66.44	52.89	−0.965
ZmJMJ31	Zm00001eb370120	401	46,042.28	5.15	48.19	82.89	−0.328
ZmJMJ32	Zm00001eb316510	362	39,915.01	5.82	56.59	81.19	−0.268
ZmJMJ33	Zm00001eb239930	127	13,834.86	5.79	41.12	88.98	−0.115
ZmJMJ34	Zm00001eb028840	118	13,036.79	6.16	56.43	80.85	−0.476
ZmJMJ35	Zm00001eb382210	405	45,232.88	6.88	52.3	80.91	−0.342
ZmJMJ36	Zm00001eb338120	243	28,026.21	6.04	44.57	87.41	−0.479

## Data Availability

The original contributions presented in this study are included in the article/Supplementary Material. Further inquiries can be directed to the corresponding author(s).

## Supplementary Materials

The following supporting information can be downloaded at https://www.mdpi.com/article/10.3390/biology15070534/s1: Table S1: Primer sequences used for qRT-PCR.
